# #MeToo in EM: A Multicenter Survey of Academic Emergency Medicine Faculty on Their Experiences with Gender Discrimination and Sexual Harassment

**DOI:** 10.5811/westjem.2019.11.44592

**Published:** 2020-02-21

**Authors:** Dave W. Lu, Michelle D. Lall, Jennifer Mitzman, Sheryl Heron, Ava Pierce, Nicholas D. Hartman, Danielle M. McCarthy, Joshua Jauregui, Tania D. Strout

**Affiliations:** * Tufts University School of Medicine – Maine Medical Center, Department of Emergency Medicine, Portland, Maine; † University of Washington School of Medicine, Department of Emergency Medicine, Seattle, Washington; ‡ Emory University School of Medicine, Department of Emergency Medicine, Atlanta, Georgia; § The Ohio State University College of Medicine, Department of Emergency Medicine, Columbus, Ohio; ¶ University of Texas Southwestern Medical School, Department of Emergency Medicine, Dallas, Texas; || Wake Forest School of Medicine, Department of Emergency Medicine, Winston-Salem, North Carolina; # Northwestern University Feinberg School of Medicine, Department of Emergency Medicine, Chicago, Illinois

## Abstract

**Introduction:**

Gender-based discrimination and sexual harassment of female physicians are well documented. The #MeToo movement has brought renewed attention to these problems. This study examined academic emergency physicians’ experiences with workplace gender discrimination and sexual harassment.

**Methods:**

We conducted a cross-sectional survey of a convenience sample of emergency medicine (EM) faculty across six programs. Survey items included the following: the Overt Gender Discrimination at Work (OGDW) Scale; the frequency and source of experienced and observed discrimination; and whether subjects had encountered unwanted sexual behaviors by a work superior or colleague in their careers. For the latter question, we asked subjects to characterize the behaviors and whether those experiences had a negative effect on their self-confidence and career advancement. We made group comparisons using t-tests or chi-square analyses, and evaluated relationships between gender and physicians’ experiences using correlation analyses.

**Results:**

A total of 141 out of 352 (40.1%) subjects completed at least a portion of the survey. Women reported higher mean OGDW scores than men (15.4 vs 10.2; 95% confidence interval [CI], 3.6–6.8). Female faculty were also more likely to report having experienced gender-based discriminatory treatment than male faculty (62.7% vs 12.5%; 95% CI, 35.1%–65.4%), although male and female faculty were equally likely to report having observed gender-based discriminatory treatment of another physician (64.7% vs 56.3%; 95% CI, 8.6%–25.5%). The three most frequent sources of experienced or observed gender-based discriminatory treatment were patients, consulting or admitting physicians, and nursing staff. The majority of women reported having encountered unwanted sexual behaviors in their careers, with a significantly greater proportion of women reporting them compared to men (52.9% vs 26.2%, 95% CI, 9.9%–43.4%). The majority of unwanted behaviors were sexist remarks and sexual advances. Of those respondents who encountered these unwanted behaviors, 22.9% and 12.5% reported at least somewhat negative effects on their self-confidence and career advancement.

**Conclusion:**

Female EM faculty perceived more gender-based discrimination in their workplaces than their male counterparts. The majority of female and approximately a quarter of male EM faculty encountered unwanted sexual behaviors in their careers.

## INTRODUCTION

Women represented 49.5% of United States (US) medical students in 2018–2019.^1^ Despite near parity in the number of men and women now entering medicine, female physicians continue to experience disparities in salary,^2^,^3^ leadership,^4^,^5^ and career advancement.^6^–^8^ For example, while 80% of the overall medical workforce is comprised of women, women hold only 13% of the healthcare industry’s executive positions.^9^ Data suggest inequity and harassment are intertwined, and harassment is often fostered in workplace environments that perpetuate these gender disparities.^10^ For instance, discrimination and harassment by gender are more prevalent in industries in which women make up a majority of the workforce but hold a minority of the positions of power.^11^ Many studies have documented gender discrimination and sexual harassment of female medical students and physicians.^5^,^12^–^19^ The recently released National Academies of Science, Engineering, and Medicine report on sexual harassment of women in medicine revealed similarly troubling results. In that report 50% of female medical students and 30% of female physicians described having been sexually harassed on the job.^20^ Inappropriate encounters were consistently reported, ranging from sexist comments and sexual innuendo to inappropriate touching and solicitation.^2^

Sexual harassment can be complex to study and measure because it has several varying legal definitions. The American Medical Association and the United Kingdom General Medical Council define sexual harassment as unwelcome attention or behavior that a person finds offensive and that makes them feel unsafe or uncomfortable.^21^,^22^ One of the more comprehensive definitions comes from the US Equal Employment Opportunities Commission (EEOC), which states that “unwelcome sexual advances, request for sexual favors, and other verbal or physical harassment of a sexual nature constitutes sexual harassment when this conduct explicitly or implicitly affects an individual’s employment, unreasonably interferes with an individual’s work performance, or creates an intimidating, hostile, or offensive work environment.”^23^ Such harassment may include unwelcome verbal, visual, non-verbal, or physical conduct that is of a sexual nature or based on someone’s gender.

There is currently little data examining gender discrimination and sexual harassment in academic emergency medicine (EM).^17^,^24^,^25^ The objective of this study was to explore the perceptions of and experiences with gender-based discrimination and sexual harassment among academic EM faculty. We hypothesized that female emergency physicians would have greater perceptions of and more experiences with gender-based discrimination and sexual harassment compared to their male colleagues.

## METHODS

### Study Design

This study was a cross-sectional survey of a convenience sample of EM faculty on their perceptions of and experiences with gender discrimination and sexual harassment in the workplace.

**Population Health Research Capsule**

What do we already know about this issue?

Female physicians experience disparities in salary, leadership, and career advancement. Prior studies have documented gender discrimination and sexual harassment of female physicians.

What was the research question?

What are the perceptions of and experiences with gender discrimination and sexual harassment among academic emergency medicine faculty?

What was the major finding of the study?

Female faculty reported more gender discrimination than male faculty, and half had encountered sexual harassment in their careers.

How does this improve population health?

There is cultural momentum to confront gender discrimination and sexual harassment across many industries. Ensuring a safe and equitable workplace is vital for the healthcare workforce.

### Study Setting and Population

All EM faculty at six urban, academic training programs were eligible for this study with the exception of the study authors. Study sites were departments of EM located in the following regions: New England (one); the Southeast (two); the South (one); the Midwest (one); the West (one). The survey was administered over February and March 2019.

### Study Protocol

An anonymous electronic survey was emailed to all eligible subjects. The invitation stated that the purpose of the study was to examine subjects’ experiences with gender discrimination and sexual harassment in their medical careers. Subjects consented to the voluntary study by completing the survey on an online, secure platform. Three reminder emails were sent to non-responders. The study was either approved or deemed exempt from review by each site’s institutional review board.

### Measurements

No single, well-validated instrument could be found that satisfactorily measured the multiple aspects of workplace gender discrimination and sexual harassment that were of interest. Based on a review of the current literature, we created a 31-item survey consisting of questions adapted from surveys used in similar work among populations of physicians from multiple specialties (Appendix). The survey was pre-tested by EM faculty at five of the six participating institutions to ensure respondent comprehension. These individuals were subsequently excluded from the study.

We measured subjects’ perceptions of discrimination using five questions adapted from the Overt Gender Discrimination at Work (OGDW) scale, an instrument that assesses the perception of gender biases in the workplace.^26^,^27^ The scale asks, “How strongly do you agree with the following statements about your current place of work:” (1) I have been treated unfairly at work because of my gender; (2) The people I work with sometimes make sexist statements and/or decisions; (3) I feel that some of the policies and practices of this organization are sexist; (4) At work, I sometimes feel that my gender is a limitation; and (5) At work, I do not get enough recognition because of my gender. Responses are based on a 1–5 Likert scale, with 1 = strongly disagree; 3 = neutral; and 5 = strongly agree. Scores range from 5–25, with higher scores indicating higher perceptions of discrimination.

Evidence supporting the reliability and validity of the OGDW when used with healthcare and other professional workers has been previously described^26^ with a Cronbach’s alpha of 0.97 and a strong, positive correlation between scores on the OGDW and another established measure of everyday gender discrimination experiences at work (*r* = 0.79; *p*<0.0001; *n*= 240).^26^ In addition, a recent study among anesthesiology trainees reported significant gender-based differences in median OGDW scores as well as in scores on the Career Barriers Inventory that reflect sexual harassment, providing further support for the construct validity for the OGDW.^27^

Using questions adapted from prior work,^14^ we also asked subjects to report the frequency with which they have *experienced* discriminatory treatment based on their gender as well as the frequency with which they have *observed* discriminatory treatment of another physician based on gender. Responses included the following: weekly, monthly, annually, rarely, and never. Those respondents who reported weekly, monthly, or annually to either experiencing discriminatory treatment or having observed discriminatory treatment were subsequently asked to identify the source of the gender-based discrimination. Potential sources included university, medical school or hospital administration, consulting or admitting physician, EM attending physician, resident physician, medical student, nursing staff, clerical staff, emergency medical services personnel, patient, and other. Subjects were asked to report the frequency with which they had experienced or had observed discriminatory treatment from each source (weekly, monthly, annually, rarely, and never). Developed by Bruce and colleagues,^14^ these items were designed to categorize the scope, type, and source of gender-based discrimination in medicine. Items were piloted with female general surgery residents and then studied in a sample of 334 female healthcare practitioners who practiced or intended to practice in general surgery. Responses to these items were consistent with qualitative responses from the same participants analyzed using a grounded theory approach. Taken together, these findings provide early evidence supporting the construct validity of the items.^14^

Lastly, we asked subjects whether in their professional career, they had encountered unwanted sexual comments, attention or advances by a work superior or colleague based on the 1980 EEOC definition of sexual harassment.^2^,^5^,^23^ For respondents who answered yes, we asked them to indicate “yes” or “no” for each of the following behaviors they may have encountered ordered by level of severity^28^: (1) sexist remarks / behavior; (2) unwanted sexual advances; (3) subtle bribery to engage in sexual behavior; (4) threats to engage in sexual behavior; (5) coercive advances; and other (we included text space to allow respondents to specify). We asked respondents who answered yes to having encountered unwanted sexual behaviors to indicate the extent to which those experiences had a negative effect on their self-confidence as a professional and on their career advancement. Both of these questions were adapted from prior work^2^,^5^ and answered via a 1–5 Likert scale, with 1 = not at all and 5 = greatly. Carr and colleagues^5^ previously showed that female medical school faculty who reported sexual harassment experiences using these items were more likely to also report gender-based bias in the academic environment, providing evidence to support the validity of these items.

We collected limited demographic information (Table 1) to prevent easy identification of otherwise anonymous responses and to encourage honest reporting. We did not obtain information linking subjects by study site.

### Data Analysis

We collected data electronically using Qualtrics (Qualtrics, Provo, UT) survey software and exported into SPSS for Windows v.25.0 (SPSS, Inc., Chicago, IL) for analysis. Continuous variables (eg, age, OGDW scores) were examined for normality using visual inspection of histograms, P-P plots, and Pearson’s skewness statistic. We used the *t*-test for independent samples to compare group means for continuous variables. In addition, we used Pearson’s chi-square analysis to compare proportions across categorical variables. In some cases, for example, in categorizing respondents as having experienced or observed gender-based discrimination, response categories were collapsed into dichotomous categories a priori to aid in result interpretation (“never” and “rarely” vs “weekly,” “monthly,” and “annually”). To assess the strength and direction of relationships between variables, we used Pearson’s correlation coefficient or Spearman’s rho as appropriate for the data. Partial correlations were also used to evaluate relationships between variables, while controlling for the effect of a covariate (gender). Data are presented as frequencies, proportions, means, and 95% confidence intervals (CI) around differences between means. All *p*-values are two-tailed, and we accepted an alpha of less than 0.05 as statistically significant.

## RESULTS

A total of 141 out of 352 (40.1%) subjects completed at least a portion of the survey. Respondents were mostly male (*n* = 80, 61.1%) and White (*n* = 104, 79.4%) (Table 1). The mean age reported by participants was 41.3 years (range 30–64 years) with the majority of respondents (*n* = 73, 55.7%) having completed residency training within 10 years.

**Figure 1:**
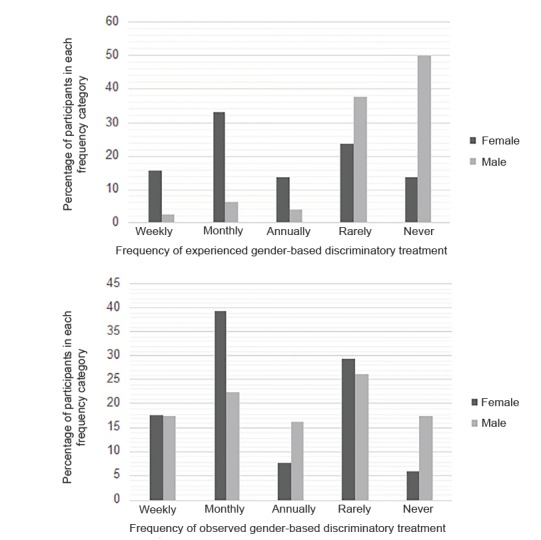
Percentage of participants who experienced or observed gender-based discriminatory treatment by gender and frequency.

In our sample, Cronbach’s alpha for the five items of the OGDW scale was 0.70, suggesting an acceptable level of internal consistency. The mean OGDW score for all respondents was 12.5 (standard deviation 4.9, 95% CI, 11.6–13.3), with women reporting significantly higher mean OGDW scores than men (15.4 vs 10.2, respectively; *t* = 6.450, *df* = 82.143, *p* < 0.001, equal variances not assumed; mean difference 5.2, 95% CI, 3.6–6.8). Female EM faculty were also significantly more likely to report having experienced workplace discriminatory treatment based on gender than their male counterparts (62.7% vs 12.5%, respectively; *p* < 0.001) (Figure 1). Having experienced discriminatory treatment based on gender was significantly associated with higher OGDW scores (mean OGDW 17.6 vs 9.8, *t* = −13.318, *df* = 87.293, *p* < 0.001; equal variances not assumed; mean difference −7.8, 95% CI, −9.0 – −6.6).

Although women were more likely than men to report having experienced gender-based discriminatory treatment, male and female EM faculty were equally likely to report having observed discriminatory treatment of another physician based on gender (64.7% vs 56.3%, respectively; *p* = 0.090) (Figure 1). Having observed discriminatory treatment of another physician based on gender was also significantly associated with higher OGDW scores (mean OGDW 14.3 vs 9.7, *t* = −6.212, *df* = 131.8, *p* < 0.001, equal variances not assumed; mean difference −4.5, 95% CI, −5.9 – −3.1). Respondent age and years in practice were not significantly correlated with OGDW scores, experience with or observations of gender-based discriminatory treatment.

**Figure 2:**
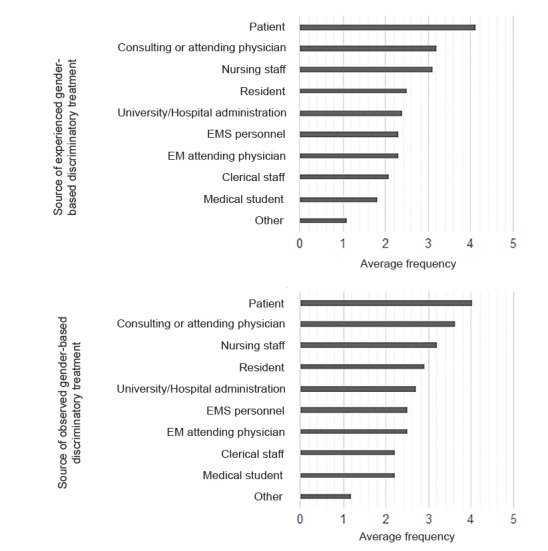
Sources of experienced or observed gender-based discriminatory treatment by average frequency. Frequency categories: 1 = never; 2 = rarely; 3 = annually; 4 = monthly; 5 = weekly.

For those respondents who had experienced or observed gender-based discriminatory treatment, at least annually, the three most frequent sources of the discriminatory treatment were patients, consulting or admitting physicians, and nursing staff (Figure 2).

The majority of women (52.9%) reported having encountered unwanted sexual comments, attention, or advanced by a work superior or colleague in their professional career (Table 2). A significantly greater proportion of women reported encountering these unwanted behaviors as compared to men (52.9% vs 26.2%, *^2^* = 9.559, *df* = 1, *p* = 0.002). The majority of unwanted behaviors were sexist remarks and unwanted sexual advances (Table 3). Of those respondents who encountered these unwanted behaviors, 22.9% (11/48) and 12.5% (6/48) reported negative effects on their self-confidence and on their career advancement at least somewhat (Table 3). Controlling for gender, those respondents who were older (*r* = 0.243, *p* = 0.011) and had been practicing longer (*r* = 0.211, *p* = 0.016) were also significantly more likely to report having encountered these unwanted behaviors. Respondents who reported having experienced these unwanted behaviors had OGDW scores that were significantly higher than those of their counterparts without such experiences (14.7 vs. 10.9, *t* = −4.516, *df* = 91.662, *p* < 0.001, equal variances not assumed; mean difference = −3.8, 95% CI, −5.4 – −2.1).

## DISCUSSION

Although gender discrimination and sexual harassment in medicine are well documented,^5^,^12^–^20^ the extent of these problems within academic EM had not been previously examined. In our study, men and women differed significantly in their perceptions of and experiences with workplace gender discrimination and sexual harassment. Our data showed that the majority of female EM faculty have encountered unwanted sexual comments, attention, or advances in the workplace. This is consistent with prior work among US medical school faculty wherein 52% of women reported harassment during their careers.^5^ A significant number of male EM faculty also reported these unwanted sexual behaviors in our study, similar to a recent study among surgery residents.^29^

It is important to note that these results spanned respondents’ professional careers, which encompass time from medical school and residency or fellowship training to their current practice as EM faculty. We did not ask respondents to identify the source of each case of unwanted sexual behavior. We therefore do not know what proportion stemmed from a work superior (eg, department chair or medical director for when respondents were faculty, or medical faculty or senior resident for when respondents were trainees) vs a work colleague (eg, peer faculty or trainee or nursing staff). Older respondents and those who have been in practice for a longer period of time were more likely to report having encountered these unwanted sexual behaviors. This is in contrast to a prior study that reported higher rates of sexual harassment among younger physicians.^16^

It is unclear in our study whether older respondents have had more time in the medical profession to encounter these behaviors, whether such behaviors were more common in the past, or whether they felt more empowered to report these instances since they may be more established in the field and have less fear of reporting. In recent work among clinician-researchers who had received career development awards from the National Institutes of Health between 2006–2009, 30% of women reported having experienced sexual harassment compared with 52% of women in the aforementioned study of medical school faculty study in 1995.^2^,^5^ While the proportion of women reporting sexual harassment appears to have decreased from 1995 to 2009, definitive conclusions cannot be drawn due to differences in study populations and the higher percentages of women enrolled in medical school in the intervening years.

Similar to other studies, the majority of unwanted sexual behaviors in our study were sexist remarks and unwanted sexual advances.^29^,^30^ Although these behaviors are detrimental and should not be tolerated, they may be less threatening than the other examples of unwanted sexual behavior included in the study survey. This may explain why a majority of respondents who described having encountered these behaviors reported that they had little to no negative impact on their self-confidence or career advancement. Our results are consistent with work among female surgeons wherein a majority similarly reported that they could overcome career barriers stemming from gender discrimination.^31^ It is important to note, however, that we do not know the cumulative impact of these less aggressive but more frequent forms of unwanted sexual behavior on individuals over the course of their professional lives. Prior research among female physicians suggested that while there were no significant differences in the effects of sexual harassment on professional confidence or career advancement, women who reported experiencing negative gender bias had lower career satisfaction.^5^ Qualitative studies of female EM faculty may be able to shed light on this important issue.

A smaller but significant number of respondents reported more alarming instances of unwanted sexual behavior, including coercive advances, bribery to engage in sexual quid pro quos, and threats to engage in sexual behavior. We did not query how respondents dealt with these unwanted behaviors, including whether they had reported them to institutional authorities or confided in mentors, colleagues, or others. Studies among surgeons found that only a minority of respondents who experienced workplace gender discrimination or sexual harassment reported it to colleagues or supervisors.^14^,^29^ The two most common reasons for non-reporting were believing that the action was harmless and that reporting would be a waste of time.^29^ Of those who reported such discrimination, a majority described a lack of action as the result.^14^

A study of internal medicine residents similarly revealed that female residents did not report harassment because they were not confident they would be helped.^18^ Among EM residents specifically, only about 3% filed a formal complaint regarding abuse or harassment.^17^ Those EM residents who did not file complaints reported a variety of reasons for not doing so, including the following: feeling that the episode was insignificant; feeling that it would not help; fear of reprisal; feeling that reporting would not stop the behavior; feeling that they had no mechanism to file; and describing that they were discouraged to report by others.^17^

Our data showed OGDW scores were significantly higher for female EM faculty than male EM faculty. Our finding was consistent with prior studies, including one among anesthesiology trainees that demonstrated a significant gender disparity in OGDW scores.^27^ In a different study, female medical school faculty were more than 2.5 times more likely than male faculty to perceive gender-based discrimination in the academic environment.^5^ Similar investigations among early-career surgery faculty and senior general surgery residents revealed that female surgeons perceived they were treated differently based on their gender and these differences in treatment were a barrier to their academic career development.^31^ As expected, our data revealed that having encountered unwanted sexual behaviors and having more experiences with and observations of gender-based discriminatory treatment correlated with higher OGDW scores.

Female EM faculty were significantly more likely to report experiencing discriminatory treatment based on their gender than their male colleagues in our study. Interestingly, male and female EM faculty were equally likely to report observing discriminatory treatment of another physician based on gender. So although someone may not have direct experience with gender discrimination, he or she can identify and recognize it when it occurs with another physician. We did not query respondents as to whether they acted or intervened in any way when they saw these instances of discrimination of another physician. Nor did we ask respondents who reported having experienced discrimination or harassment whether others intervened on their behalf when there were witnesses. Institutional policies and guidance illustrating how witnesses should report and intervene in instances of gender discrimination or sexual harassment may be helpful.

EM faculty reported that patients were the most common source of both experienced and observed gender-based discriminatory treatment. This may stem from underlying sexist beliefs that exist within our culture and society. Prior qualitative work revealed that despite the power physicians hold in the relationship with their patients, it did not preclude female physicians from being the target of unwanted sexual harassment and sexual advances.^32^ In these circumstances, female physicians were viewed as women first and physicians second, leaving them susceptible to sexual harassment, particularly by male patients. Physicians described sexual harassment from patients most commonly in the form of suggestive looks or gestures and sexual remarks.^19^

Among EM residents, women were more likely to report unwanted sexual advances and discomfort from sexual humor, and that patients or patients’ family members were the most frequent source of abuse or harassment.^17^.^25^ To be clear, in our study we only asked respondents about discriminatory behavior, not harassment, from patients. Nonetheless, significant overlap exists between the two types of behavior and there is evidence to suggest that progress has been limited. In a recent study of female medical students, all participants reported numerous workplace interactions with male patients involving flirting or sexual innuendo, with many describing that they were “too used to it.”^12^

The second and third most common sources of experienced and observed gender-based discriminatory treatment were consulting or admitting physicians and nursing staff. This is consistent with prior work among surgery residents, where among all hospital staff, nurses were the most common perpetrators of harassment, followed by attending physicians.^29^ Sexism within the medical profession is well documented, starting from undergraduate medical education, through residency and fellowship training, and continuing through clinical practice as attendings.^14^ In a recent study investigating the prevalence of sexual harassment in academic medicine, the presence of a strong institutional hierarchy was associated with sexual harassment in both genders, highlighting the important role of organizational culture.^30^

While issues related to gender discrimination and sexual harassment in medicine have long been documented, there is currently significant societal and cultural momentum to confront these pervasive problems. Prominent attention to sexual harassment and assault has been raised through the #MeToo movement, which aims to shed light on the prevalence of sexually inappropriate behaviors. The #MeToo movement subsequently spurred the TIME’S UP organization that coordinates responses and develops solutions to address gender discrimination and harassment. TIME’S UP Healthcare was recently established to unify national efforts to bring safety, equity, and dignity to the healthcare workplace.^33^

There are many ways gender-based discrimination and sexual harassment can be addressed. For example, leaders in medicine can commit to ending gender-based inequities by changing workplace standards and culture. Medical educators can better prepare students, residents, and fellows for dealing with gender-based discrimination and sexual harassment in their present role as trainees and future role as physicians. Physicians should also take advantage of their inherent leadership roles in healthcare and advocate for each other as well as other healthcare providers who may not feel empowered to speak up. Future research examining and describing successful strategies (eg, staff education, clear anti-harassment policies, reliable reporting mechanisms, strict accountability, changes to academic promotion processes, and faculty recruitment and retention) to address gender inequities and sexual harassment in the healthcare workplace is necessary.^29^

## LIMITATIONS

Our study population was a convenience sample of EM faculty at six urban academic sites and our results may not be generalizable to practicing emergency physicians in non-urban and non-academic settings. Approximately 40% of eligible subjects responded to the survey and response bias may have played a role in our results. We were unable to compare characteristics of respondents with those of non-respondents due to the anonymous nature of our survey methodology. Therefore, we do not know whether more men or women chose to participate in the study and whether their experiences with gender discrimination or sexual harassment played a role in their study participation.

Although our questions measuring self-reported experiences and observations of gender discrimination and unwanted sexual behavior were modeled after prior work, have face validity as well as internal consistency reliability (ɑ = 0.70) in this sample, other aspects of reliability and criterion and construct validity have not been previously established

Finally, we were unable to corroborate respondents’ self-reported experiences with and observations of gender discrimination or sexual harassment. Prior work demonstrated that the majority of medical students developed progressive desensitization to discrimination and learned to systematically tolerate or minimize discrimination or harassment as a part of their future career.^12^ Thus, we do not know whether respondents’ accounts of experienced or observed gender discrimination and sexual harassment represent over- or under-reporting of what may be considered objective definitions of discrimination or harassment.

## CONCLUSION

Female EM faculty perceived more gender-based discrimination in their workplace than their male counterparts, with higher perceptions of discrimination associated with greater reports of experience with and observations of discriminatory treatment. Although female EM faculty were more likely to experience gender discrimination than their male colleagues, both groups were similar in their observations of discriminatory treatment of another physician based on gender. The majority of female and approximately a quarter of male EM faculty encountered unwanted sexual comments, attention, or advances by a work superior or colleague during their professional careers. Future work to examine the prevalence and characteristics of gender discrimination and sexual harassment in a larger and more diverse sample of emergency physicians is necessary.

## Supplementary Information



## Figures and Tables

**Table 1:** Characteristics of participants in survey of gender bias and sexual harassment

Characteristics	Participants (N=141) n (%)
Age years)	
<39	52 (47.3)
40–49	41 (37.3)
50–59	16 (14.5)
≥60	1 (0.9)
Years out of training	
1–5	33 (25.2)
6–10	40 (30.5)
11–15	26 (19.8)
16–20	15 (11.5)
≥21	17 (13.0)
Gender	
Male	80 (61.1)
Female	51 (38.9)
Race/ethnicity	
White	104 (79.4)
Black/African American	6 (4.6)
Hispanic/Latino	5 (3.8)
Asian/Pacific Islander	12 (9.2)
American Indian/Alaska Native	2 (1.5)
Other	2 (1.5)

**Table 2:** Number of participants by gender who reported having encountered unwanted sexual comments, attention, or advances.

Response	Female n (%)	Male n (%)	Total n (%)
No	24 (47.1%)	59 (73.8%)	83 (63.4%)
Yes	27 (52.9%)	21 (26.2%)	48 (36.6%)

**Table 3:** Type and impact of unwanted sexual comments, attention, or advances.

Action type	Total n (%)
Sexist remarks/behavior	45 (48.4)
Unwanted sexual advances	36 (38.7)
Coercive advances	8 (8.6)
Subtle bribery to engage in sexual behavior	3 (3.2)
Threats to engage in sexual behavior	1 (1.1)
Extent these behaviors had a negative effect on your confidence in yourself as a professional	
Greatly	4 (8.3)
Moderately	1 (2.1)
Somewhat	6 (12.5)
A little	3 (6.3)
None at all	34 (70.8)
Extent these behaviors negatively affected your career advancement	
Greatly	1 (2.1)
Moderately	2 (4.2)
Somewhat	3 (6.2)
A little	7 (14.6)
None at all	35 (72.9)
