# Supplementary material for: #MeToo in EM: A Multicenter Survey of Academic Emergency Medicine Faculty on Their Experiences with Gender Discrimination and Sexual Harassment
Source: West J Emerg Med. 2020 Feb 21;21(2):252–60. doi: 10.5811/westjem.2019.11.44592 (PMC7081862; doi:10.5811/westjem.2019.11.44592)
Supplement: Supplementary file 1 [file wjem-21-252-s001.docx]

**Appendix: Survey Questions Related to Gender Discrimination and Sexual Harassment**

Gender Discrimination Questions

How strongly do you agree with the following statements about your current place of work? (scale of 1-5, with 1 = strongly disagree, 3 = neutral, 5 = strongly agree)

1. I have been treated unfairly at work because of my gender.
2. The people I work with sometimes make sexist statements and/or decisions.
3. I feel that some of the policies and practices of this organization are sexist.
4. At work, I sometimes feel that my gender is a limitation.
5. At work, I do not get enough recognition because of my gender.

----------

1. In my current institution, I’ve experienced discriminatory treatment based on my gender:

- Weekly
- Monthly
- Annually
- Rarely
- Never

If Weekly, Monthly, Annually:

6.A. How often have you experienced discriminatory treatment based on your gender from the following sources?: (for each source, choice of weekly, monthly, annually, rarely, never)

- University / Medical School or Hospital administration
- Consulting or admitting physician
- EM attending physician
- Resident
- Medical student
- Nursing staff
- Clerical staff
- EMS personnel
- Patient
- Other: __________

1. In my current institution, I’ve observed discriminatory treatment of another physician based on gender:

- Weekly
- Monthly
- Annually
- Rarely
- Never

If Weekly, Monthly, Annually:

7.A. How often have you observed discriminatory treatment of another physician based on gender from the following sources?: (weekly, monthly, annually, rarely, never)

- University / Medical School or Hospital administration
- Consulting or admitting physician
- EM attending physician
- Resident
- Medical student
- Nursing staff
- Clerical staff
- EMS personnel
- Patient
- Other: __________

1. In your professional career, have you encountered unwanted sexual comments, attention or advances by a work superior or colleague?

- No
- Yes

If yes:

8.A. Please indicate “yes” or “no” for each of the following that you may have encountered:

- - Sexist remarks/behavior
  - Unwanted sexual advances
  - Subtle bribery to engage in sexual behavior
  - Threats to engage in sexual behavior
  - Coercive advances
  - Other (please specify)

8.B. To what extent have these experiences had a negative effect on your confidence in yourself as a professional? (1-5 Likert scale, 1 = not at all, 5 = greatly)

8.C. To what extent have these experiences negatively affected your career advancement? (1-5 Likert scale, 1 = not at all, 5 = greatly)

Demographics

1. What is your age?
2. How many years have you been practicing since completion of residency?

- 1-5 (=1)
- 6-10 (=2)
- 11-15 (=3)
- 16-20 (=4)
- 21+ (=5)

1. What is your race or ethnicity? (check all that apply)
   - - American Indian / Alaska Native (=1)
     - Asian (=2)
     - Black / African American (=3)
     - Hispanic / Latino (=4)
     - Native Hawaiian / other Pacific Islander (=5)
     - White (=6)
     - Other (please specify) (=7)
2. What is your gender identity?

- Male (=1)
- Female (=0)
- Transgender
- Another gender identity
